# Real-time co-site optical microscopy study on the morphological changes of the dentine’s surface after citric acid and sodium hypochlorite: a single-tooth model

**DOI:** 10.1186/s12903-021-01815-6

**Published:** 2021-09-17

**Authors:** Wojciech Wilkoński, Lidia Jamróz-Wilkońska, Szczepan Zapotoczny, Janusz Opiła, Luciano Grandino

**Affiliations:** 1 Research Department of the Polish Endodontic Association, Kielce, Poland; 2 Faculty of Chemistry of the Jagiellonian University, ul. Gronostajowa 2, 30-387 Kraków, Poland; 3 Chair of Applied IT of the Faculty of Management of AGH University of Science and Technology, Kraków, Poland; 4 Crotone, Italy

**Keywords:** Sodium hypochlorite, Citric acid, Root canal, Irrigation, Smear layer, Demineralization, Dentine

## Abstract

**Background:**

To this day, the effects of sodium hypochlorite and chelating agents on the smear layer and on the dentine’s surface, remain not fully examined. The study is aimed to analyze the dentine's surface treated with 40% citric acid and 5.25% sodium hypochlorite according to two irrigation protocols.

**Materials and methods:**

The study employed a computer-controlled Nikon Eclipse LV100 optical microscope. Ten roots split longitudinally with canals prepared mechanically using the MTwo system to a size of 40/04 were observed. The root halves were divided into two study groups, one half in each of the groups. According to two irrigation protocols, the dentine's surface was irrigated with 40% citric acid and 5.25% sodium hypochlorite, separated with water. Dentine surface was observed in a fixed place and photographed in 500 × magnification after each irrigation stage. The obtained images were then analyzed using computer software (NIS-Elements AR, GIMP-2.6, ImageJ 1.45s).

**Results:**

Various speed of removal of the smear layer and varied morphological changes of the dentine's surface were observed in both examined groups. Double irrigation of the dentine with citric acid for 30 s resulted in complete removal of the smear layer, and double irrigation for 60 s resulted in increased mean diameter of the dentine tubules (degradation of the dentine's surface).

**Conclusions:**

Double alternating irrigation with citric acid and sodium hypochlorite for 30 s yielded satisfactory results, while double irrigation for 60 s resulted in increased mean diameter of the dentine tubules. A real time assessment of the dentine’s surface changes after both tested solutions can improve understanding of the phenomena that occur during the irrigation and as a result it might help to improve clinical outcomes in root canal treatment.

**Supplementary Information:**

The online version contains supplementary material available at 10.1186/s12903-021-01815-6.

## Background

During the endodontic treatment, the smear layer is created due to the mechanical preparation of root canals. It covers canal walls and closes dentinal tubules [1]. Smear layer removal of the from the root canal is controversial. On the one hand, removing the smear layer increases dentine's permeability and, as a result, penetration of antiseptics and sealers for dentinal tubules. On the other hand, however, it causes changes in the chemical composition and physical properties of the dentine. Chelating agents are commonly used for the removal of the inorganic component of the smear layer. They also cause demineralization of the dentine, resulting in exposure of collagen fibers and dentinal surface erosion. Uneven dentine's surface has a negative effect on the adaptation of the material obturating root canals, and the collagen matrix is not thoroughly hybridized with sealers [2, 3]. The resulting micro-and nano-leakage can potentially negatively affect the results of endodontic treatment [4].

The majority of academic papers concerning the surface of root canal walls are based on studies using a Scanning Electron Microscope (SEM). Such studies have numerous flaws: a specimen is processed with chemicals, dehydrated with alcohol, and coated with a nanoscale layer of metal, then observed in a high vacuum. Hydrated organic structures collapse during dehydration of specimens for testing [4]. De-Deus et al. described test methods using an optical microscope as an alternative to SEM tests [5–7]. Such tests provide an image of the sample surface in a water environment without damaging the sample. The samples can be observed during irrigation, obtaining real-time images. Concerning such possibilities, an experiment was carried out to analyze changes in the dentine's surface caused by irrigation fluids commonly used in endodontics.

## Methods

According to two irrigation protocols, the study aimed to analyze the dentine's surface immediately after irrigation with citric acid and sodium hypochlorite.

The study included ten human upper canines removed due to periodontal problems. The teeth after extraction were stored in 1% solution of chloramine. After cleaning the roots, anatomical crowns were cut off using drills with diamond coating and water–air cooling. Root canals were prepared using the Mtwo system to a size of 40 taper 04 using „crown-down” technique following with the previously described sequence [8]. Following preparation of the root canals, the canals were irrigated with 5.25% sodium hypochlorite for 1 min. The roots were then incised longitudinally using discs with double-sided diamond coating and constant water–air cooling, then split with a chisel into two halves with similar sizes. The halves were then divided into two study groups. The roots were placed on the platform of Nikon Eclipse LV100 (Nikon, Japan) optical microscope one by one and central parts of root canals in the most representative spot were observed in 500 × magnification. Output image of the root surface was recorded before irrigation. Maintaining the same position of specimens, the canals were irrigated according to the following protocols:

- Group 1:
  - Stage 1: 30 s—40% citric acid,
  - Stage 2: 30 s—5.25% sodium hypochlorite,
  - Stage 3: 30 s—40% citric acid,
  - Stage 4: 60 s—5.25% sodium hypochlorite,
- Group 2:
  - Stage 1: 60 s—40% citric acid,
  - Stage 2: 30 s—5.25% sodium hypochlorite,
  - Stage 3: 60 s—40% citric acid,
  - Stage 4: 60 s—5.25% sodium hypochlorite,

After each subsequent irrigation stage, the canals were thoroughly washed with distilled water in order to stop chemical reactions, and then immediately observed in 500 × magnification.

### Obtaining images and measurements

Each specimen was observed before recording images and conducting the study. Each observation with manual adjustment of the microscope platform was aimed at determination of limit points of the distance between the specimen and the lens ensuring sharp image of the specimen. Such parameters were then entered into to the computer software NIS-Elements AR (NIS-Elements, USA). A computer-controlled digital camera took 30–90 pictures in 2560 × 1920 resolution with moving the sample away from the lens in 0.5 µm increments within the determined limits. The aggregate images of the canal dentine’s surface were obtained by superimposing several dozens of images with a total resolution of 0.14 µm/pixel, whereas the computer software used the algorithm for selective superimposition of fragments of images with sharp contours. The obtained images were saved to graphic file formats and subject to further processing and measurement using GIMP-2.6 (GIMP Development Team) and ImageJ 1.45s (National Institutes of Health, USA) software done by blinded observer (WW) after encoding data. For the purposes of the analysis, the files were converted to 8-bit version, then binarized so that the open dentinal tubules were visible as circles and ovals. The following parameters were analyzed and measured:

- changes in the diameter of open dentinal tubules,
- effectiveness of smear layer removal.

Diameters of dentinal tubules were determined on the basis of measurements in ImageJ 1.45s application. Diameters of all the dentinal tubules, whose full circumference was contained in the area of 100 × 100 µm in the central point of the image, were calculated. The averaged values together with standard deviation were taken into consideration in the statistical analysis.

The smear layer removal was evaluated upon the measurement of the percentage of opened dentinal tubules measured via software (ImageJ). A five-degree scale was used to assess the efficiency of smear layer removal:

- 1—no smear layer—all the dentinal tubules were open,
- 2—a small area covered with the smear layer (> 75% of the dentinal tubules were open),
- 3—the smear layer partially covering the dentine (50–75% of the dentinal tubules were open),
- 4—the smear layer covering the dentine to a large extent (10–49% of the dentinal tubules were open),
- 5—the smear layer completely covering the dentine (< 10% of the tubules were open).

The data was gathered in a database, decoded (after evaluation by blinded observer) and subjected to statistical analysis using the following tests: Kruskal–Wallis’ test, sign test and U Mann–Whitney’s test, with statistical significance set at *p* ≤ 0.05 (Table 1).

**Table 1:** Statistical tests used to calculate the significance of the differences between both experimental groups

Test used	Parameter tested	Stage 1 Gr 1 vs Gr 2		Stage 2 Gr 1 vs Gr 2		Stage 3 Gr 1 vs Gr 2		Stage 4 Gr 1 vs Gr 2		Stage 3 Gr 1 vs Stage 1 Gr 2	
		*p*	Z	*p*	Z	*p*	Z	*p*	Z	*p*	Z
Sign test	Changes of the diameters of dentine tubules	0.0044	2.846	0.0044	2.846	0.0044	2.846	0.0044	2.846		
Mann–Whitney U										0.00	− 3.743
Mann–Whitney U	Smear layer removal efficiency	0.143	1.629	0.393	0.981	0.739	0.453	1.00	− 0.073	0.024	2.258
Kruskal–Wallis		0.131		0.364		0.705		1.00			

## Results

In group 1, following the first stage of irrigation (citric acid for 30 s), no effective removal of the smear layer was observed—10% of the canals were cleaned to the degree 1, 50%—degree 2 and 40%—degree 3 and 4. In group 2, after the first irrigation stage (citric acid for 60 s), 30% of the canals were cleaned to the degree 1, 60%—to the degree 2 and 10%—degree 3. Differences in the degree of removal of the smear layer between the groups after the first stage of irrigation were not statistically significant (*p* = 0.13). However, statistically significant differences in the diameter of the dentinal tubules were identified (*p* = 0.004). After the second stage of irrigation (sodium hypochlorite for 30 s), improved degree of removal of the smear layer from the canal walls was observed in both groups. In group 2 canals, there were more canals with the smear layer removed than in group 1, however, the differences were not statistically significant (*p* = 0.36). Also, in both groups, an increase of the diameter of dentinal tubules was observed and such changes were statistically significant (*p* = 0.004). After the third irrigation stage (citric acid for 30 s in group 1 and 60 s in group 2), further improvement of the smear layer removal and a further increase in the dentine tubules' diameter were observed. No statistically significant differences were observed between the two groups concerning smear layer removal (*p* = 0.71), however, the differences in diameters of the dentinal tubules between the groups were statistically significant (*p* = 0.004). Statistically significant differences between group 1 after the third irrigation cycle and group 2 after the first irrigation cycle were observed (total irrigation time with citric acid—60 s) both with regard to the level of smear layer removal and diameters of the dentinal tubules (*p* = 0.02; *p* = 0.00, respectively). After the fourth irrigation stage (sodium hypochlorite for 60 s), the same percentage of canals with smear layer entirely removed. An increase of the diameters of dentinal tubules was observed in both groups. In contrast, in group 2, the dentinal tubules' diameter was statistically significantly larger (*p* = 0.004). The data have been presented in Tables 1, 2 and 3.

**Table 2:** Diameters [μm] of dentine tubules (mean ± SD) in experimental groups after four irrigation stages

	Stage 1	Stage 2	Stage 3	Stage 4
Group 1	3.68 ± 0.06	4.13 ± 0.06	4.35 ± 0.05	5.10 ± 0.08
Group 2	3.92 ± 0.06	4.78 ± 0.08	4.97 ± 0.08	5.82 ± 0.10

**Table 3:** Smear layer scores in group 1 and 2 before and after four irrigation stages

	Stage 0		Stage 1		Stage 2		Stage 3		Stage 4	
	Gr 1	Gr 2	Gr 1	Gr 2	Gr 1	Gr 2	Gr 1	Gr 2	Gr 1	Gr 2
Score 1	0	0	1	3	4	6	7	8	9	9
Score 2	0	0	5	6	5	4	3	2	1	1
Score 3	0	0	3	1	1	0	0	0	0	0
Score 4	0	0	1	0	0	0	0	0	0	0
Score 5	10	10	0	0	0	0	0	0	0	0

## Discussion

Removal of the smear layer is a commonly recommended procedure. However, no evidence could prove that removing the smear layer is necessary for better results of endodontic treatment. The abundance of factors affecting the treatment results makes it very difficult to obtain statistically significant differences in such clinical studies. The primary goal of endodontic treatment in infected teeth is to remove pathogens and the complete root canals obturation. The studies show that pathogens can penetrate dentinal tubules up to 200 µm [9]. Therefore, removing the smear layer is theoretically necessary to penetrate antiseptic agents and sealers into dentinal tubules.

Smear layer removal is a chemical reaction that takes place in a given time and space of the endodontic system. Recording phenomena related to such process is usually connected with the use of scanning electron microscopes (SEM). Preparation of SEM examination specimens consists of their gradual dehydration in a graded ethanol solution and coating them with a layer of precious metals. Then, samples are observed in high vacuum. Such specimens, as hydrated spatial structures, change both in volume and morphology. Proteins become denaturated with alcohol and collapse due to desiccation. Such images do not reflect subtle details of the dentine’s surface and can lead to formulation of erroneous conclusions. Tay et al. modified the method of preparation of specimens by using silazane before application of metal in order to fill in spaces left after water [3]. As a result of such modification, images that differ significantly from the previously observed ones were obtained. Probably what had been considered dentine is actually collapsed collagen coated with metal. Therefore, the previous studies presenting alleged dentinal erosion resulting from treatment with sodium hypochlorite after chelating agents, actually present the image of the dentine after dissolution of exposed collagen [3]. This study also revealed changes in diameters of dentinal tubule orifices due to of lysis of the collagen with sodium hypochlorite. In other studies, Tay et al. proved that exposed collagen could be a potential source of problems as it cannot be thoroughly infiltrated with sealers. Tay et al. assumed that the use of hypochlorite after chelating agents can be beneficial for nano-sealing ability, as application of chelating agents causes erosion of the dentine anyway [2].

Despite the progress in methodology and extreme precision of SEM examinations, real-time observation of processes is impossible. A specimen prepared for SEM examination is destroyed and its further chemical processing, examination, observation and comparative analysis before and after processing are not possible. Therefore, it is necessary to prepare specimens after each cycle/method of irrigation. De-Deus et al. used an Atomic Force Microscope (AFM) to assess smear layer removal speed in real-time [10]. AFM examination provides an image of the sample surface in water environment without damaging the specimen. The same surface can be rinsed and analyzed again. The authors identified limitations of such method, mainly related to the scanning time of the specimen. In other studies, De-Deus et al. used a computer-controlled optical microscope and digital image processing [5–7]. This study employs optical microscope as well. Computer control and software ensured sharp images despite large differences in the distance between individual parts of canal walls and the lens. Similarly to De-Deus et al., this study uses two halves of the same tooth divided into two study groups (single tooth method) [6]. It ensures very similar qualitative distribution of specimen’s dentine in the study groups.

This study employs a complex, five-degree scale assessing the effectiveness of smear layer removal, eliminating any possible contribution of subjective human nature. Precise measurements using computer software and division concerning the percentage share of open tubules also allow for assessment of the speed of smear layer removal after given irrigation cycles.

**Fig. 1:**
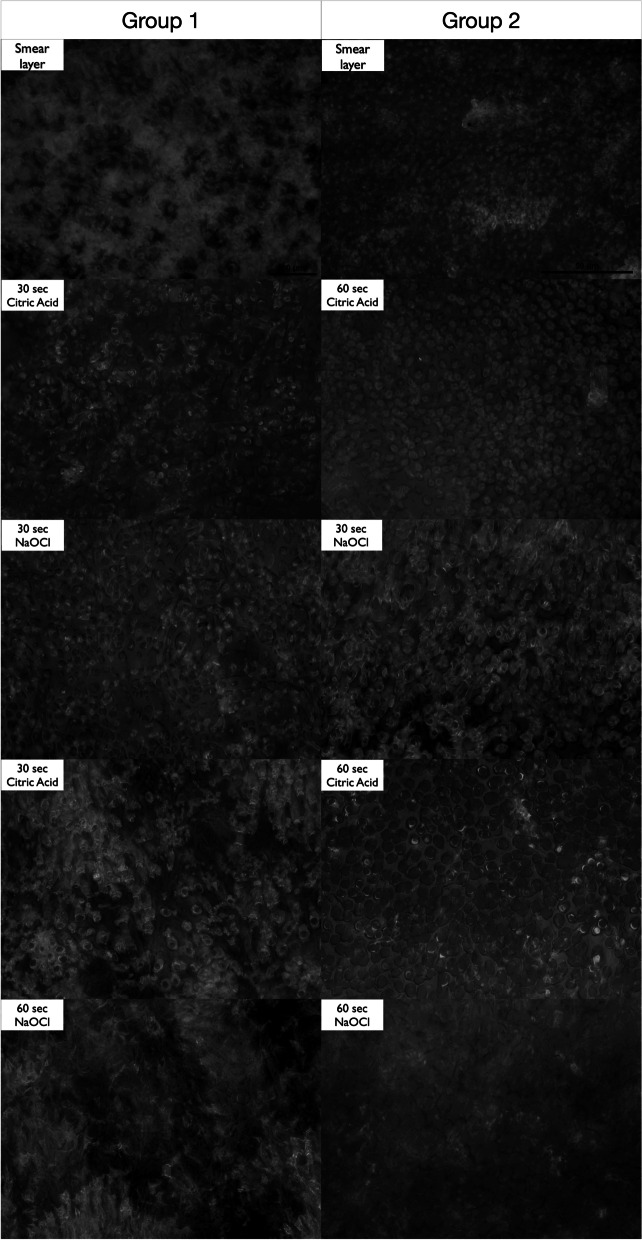
Surface changes of dentine after four stages within two tested irrigating protocols. Two columns show samples obtained from the same split tooth: left column represents group 1, right—group 2. Images of the same region of the root canal show the progress of demineralization and degradation

The studies show that the concentration of citric acid affects the speed of demineralization and smear layer removal effectiveness. In the study by Prado et al., 10% concentration was applied for 30, 60 and 180 s with better results in comparison with 17% EDTA [1]. However, Khedmat et al., also using 10% concentration of citric acid, obtained worse efficiency in the apical section than with 17% EDTA [11]. In this study, a 40% concentration with a total irrigation time of 60 and 120 s was used. No statistically significant changes in the eventual effectiveness of smear layer removal were found in both study groups. In the group irrigated with citric acid in two 60-s cycles, faster smear layer removal was observed. After the first 60-s cycle, the smear layer's reduction was identified in 30% of the specimens. In comparison, in group 1, after the first 30-s cycle, the smear layer was removed in 10% of the samples. In both study groups, alternate irrigation with sodium hypochlorite dissolved demineralized tissue, increasing diameters of dentinal tubule orifices, and probably improved acid penetration in the following irrigation cycle. A higher percentage of canals with removed smear layer in group 1 after the third stage of irrigation in comparison with group 2 after the first irrigation stage (total time of citric acid activity—60 s) shows an improvement of the effectiveness of citric acid after the prior dissolution of organic compounds with sodium hypochlorite. In the second group, double 60-s irrigation with citric acid caused degradation of the dentine's surface reflected in the distortion of homogeneous structure and erosion of the tubules' edges. (Fig. 1). Therefore, despite limitations of the study, it can be concluded that double irrigation with citric acid after 30 s and 60 s was equally effective in removing the smear layer, and irrigation in two cycles 60 s each can result in increased diameter of the dentinal tubules, which might be perceived as undesired morphological changes on the dentine's surface. Such assumptions require further studies using different methodologies and imaging methods, considering limitations of fluid distribution and the varied dentine structure in individual parts of root canals.

Despite of the efforts, this experiment has limitations. There is a potential risk of sampling and distribution errors that can lead to distorted assessment of the results. This study is focused in the measurements (of the smear layer removal and diameter of the dentine’s tubules) only in selected, flat regions of the root canal walls, not the entire the root canal. Thus, the gathered data may not reflect the true results. In this experiment, in order to achieve the precision, sensitivity and repeatability of the measurements, only straight root canals with simple anatomy were used. The outcomes in curved or oval/flat/irregular root canals could be different. It should be also pointed out that the small sample size, while is sufficient enough to gain satisfactory laboratory results, may not be fully representative. It should be mentioned that this study cannot be directly referred to as a clinical situation because, during the irrigation of root canals, the fluid is administered with a needle and not perpendicularly to the dentine's surface (Additional file 1).

## Conclusions

Upon this in vitro study, with its shortcomings, it may be concluded that double alternating irrigation with citric acid and sodium hypochlorite is an effective way of removing the smear layer. Cycles of 30 s each were sufficient to gain satisfactory results, while 60 s ones resulted in increased mean diameter of dentine tubules.

## Supplementary Information

**Additional file 1.** Detailed data is available in the additional file upon reasonable request (Additional file 1).

## Data Availability

The datasets used and/or analysed during the current study are available from the corresponding author on reasonable request.

## Abbreviations

AFM: Atomic force microscope
EDTA: Ethylenediaminetetraacetic acid
SEM: Scanning electron microscope
