# Correction: Real-time co-site optical microscopy study on the morphological changes of the dentine’s surface after citric acid and sodium hypochlorite: a single-tooth model

**DOI:** 10.1186/s12903-022-02437-2

**Published:** 2022-09-15

**Authors:** Wojciech Wilkoński, Lidia Jamróz-Wilkońska, Szczepan Zapotoczny, Janusz Opiła, Luciano Giardino

**Affiliations:** 1 Research Department of the Polish Endodontic Association, Kielce, Poland; 2 Faculty of Chemistry of the Jagiellonian University, ul. Gronostajowa 2, 30‑387 Kraków, Poland; 3 Chair of Applied IT of the Faculty of Management of AGH University of Science and Technology, Kraków, Poland; 4 Crotone, Italy

## Correction to: BMC Oral Health (2021) 21:454 https://doi.org/10.1186/s12903-021-01815-6

In this article, the author’s name Luciano Giardino was incorrectly written as Luciano Grandino. This has been corrected with this correction.

The original article has been corrected.

